# Enhanced blood glucose levels prediction with a smartwatch

**DOI:** 10.1371/journal.pone.0307136

**Published:** 2024-07-18

**Authors:** Sean Pikulin, Irad Yehezkel, Robert Moskovitch

**Affiliations:** Software and Information Systems Engineering, Ben Gurion University of the Negev, Beer Sheva, Israel; St Xavier’s Catholic College of Engineering, INDIA

## Abstract

Ensuring stable blood glucose (BG) levels within the norm is crucial for potential long-term health complications prevention when managing a chronic disease like Type 1 diabetes (T1D), as well as body weight. Therefore, accurately forecasting blood sugar levels holds significant importance for clinicians and specific users, such as type one diabetic patients. In recent years, Continuous Glucose Monitoring (CGM) devices have been developed and are now in use. However, the ability to forecast future blood glucose values is essential for better management. Previous studies proposed the use of food intake documentation in order to enhance the forecasting accuracy. Unfortunately, these methods require the participants to manually record their daily activities such as food intake, drink and exercise, which creates somewhat inaccurate data, and is hard to maintain along time. To reduce the burden on participants and improve the accuracy of BG level predictions, as well as optimize training and prediction times, this study proposes a framework that continuously tracks participants’ movements using a smartwatch. The framework analyzes sensor data and allows users to document their activities. We developed a model incorporating BG data, smartwatch sensor data, and user-documented activities. This model was applied to a dataset we collected from a dozen participants. Our study’s results indicate that documented activities did not enhance BG level predictions. However, using smartwatch sensors, such as heart rate and step detector data, in addition to blood glucose measurements from the last sixty minutes, significantly improved the predictions.

## Introduction

Predicting Blood Glucose (BG) levels is desirable for various purposes, such as for type 1 diabetes patients [1], as well as for professional athletes [2]. Knowing in advance when BG is approaching unsafe levels, whether low or high, provides time to proactively avoid hypo\hyper-glycemia and their associated complications. The management of diabetes has become easier in recent years due to advancements in blood glucose (BG) monitoring technology [3]. Continuous glucose monitoring (CGM) devices enable patients to measure their blood glucose (BG) levels almost in real-time. The glucose data accessible through CGM devices, coupled with the push to perfect an artificial pancreas [4] has spurred increased interest in utilizing machine learning (ML) and artificial neural networks (ANN) approaches to enhance prediction accuracy [5]. The BG levels are affected by daily activities, such as food and drink intake, and exercises combined with physiological data such as the heart rate (HR) [6]. In recent studies, the authors tried to improve the prediction by take in account the user’s daily activities [7, 8]. In order to obtain daily activities data, the participants were required to record their daily activities manually. However, the main problem with such approaches is that the manual recording makes it difficult to incorporate in the daily life of the participants and worse—leads to inaccurate documented data. To overcome this problem, we propose tracking the participant’s hand movements using a smartwatch. The smartwatch’s sensors are continuously being recorded and analyzing the sensors data using machine and deep learning methods. Our method does not require documentation and requires minimal hardware while at the same time it achieves very high predictive accuracy. The proposed method includes automatically collecting the data from the participates and predicting BG levels according to the current BG together with the smartwatch sensors values. We evaluated the method on a dataset that consisted on monitoring twelve participants which each of them wore our dedicated smartwatch and CGM for 10 days.

This work aims to create an accurate personal prediction model for each participant that utilizes CGM data and automatically monitored sensors’ data. The goal is to remove completely the use of manual activities and to utilize commonly used technological devices to gain better predictions.

The main contributions of the paper are the following: 1. A novel approach for human’s blood glucose levels forecasting, which is enhanced by wearable device’s sensory data, such as step detector, heart rate, in addition to the glucose monitoring readings. 2. A rigorous evaluation on real data of a dozen of participants.

## Related work

### Blood glucose levels

Blood glucose (BG) levels refer to the concentration of glucose circulating in the bloodstream. Glucose, a fundamental sugar molecule, typically amounts to around 4 grams within the blood of a 70-kilogram human [9]. The BG levels are regulated as part of the body’s metabolic homeostasis. For individuals without diabetes, the standard fasting BG goal falls within the range of 3.5–5.5 mmol/L (65-100 mg/dl) [10] and is critical for normal function in a few tissues. Glucose levels are usually particularly low in the morning, before eating breakfast, or high after various meals. Glucose is transported across the intestinal wall, entering the hepatic portal vein before reaching liver cells and various other tissues [11]. The pancreas maintains blood glucose levels within the normal range through its various hormones, particularly glucagon and insulin [12]. BG levels outside the normal range may be an indication for medical complications indicator of diabetes [13].

Diabetes is a chronic condition which leads to difficulties in regulating BG levels. This condition arises from two primary causes. The first, known as Type 1 diabetes involves the body’s inability to produce insulin and the second is Type 2 diabetes, in which the body can’t efficiently utilize the insulin it produces [14]. T1D is characterized by immune-mediated destruction of *β*-cells in the pancreas by T cells of the immune system. This destruction reduces the ability of the pancreas to produce sufficient insulin [15]. The prevalence of T1D has been consistently rising worldwide (3% per year) [16]. Subcutaneous insulin therapy stands today as the the only treatment for T1D, and it requires knowledge of the exact insulin dose to prevent both hypoglycemia and hyperglycemia. Hypoglycemia is a term that refers to a situation of abnormally low levels of glucose (usually below 70 mg/dL). The common symptoms of hypoglycemia are shakiness, confusion, sweating, and, if severe, loss of consciousness [17, 18]. Hyperglycemia is a term used to describe a situation of high levels of gluocse in the blood plasma (usually above 180 mg/dL). The common symptoms are increased thirst and a dry mouth, tiredness and urinating large amounts. This condition can also affect other systems in the human’s body and lead to complications such as kidney disease [19], and cardiovascular disease [20]. Consequently, achieving an optimal injection dosage is crucial for ensuring proper healthcare. Hence, CGM is designed to provide a more complete picture and the use of it can facilitate improved treatment decisions and enhanced glucose management. The continuous glucose data provided by CGM can be used by prediction models, enabling the creation of a system capable of predicting BG levels and issuing alerts it there is a risk of hypoglycemia or hyperglycemia.

#### Predicting blood glucose levels

In recent years, some studies had tried to estimate upcoming values of BG levels, using ML techniques. Accurate estimators for prediction, even of short horizons, may allow reducing the risks of serious life-threatening complications, including stroke and heart disease [21]. Pappada et al. [22] utilized a Neural Network-based model to predict BG levels. The model integrated manually recorded data of food intake, insulin and physical activity, in order to increase the precision of the BG level estimation. While this model accurately predicted hyperglycemic episodes, it failed to predict hypoglycemic episodes, a possible reason for that is the lesser occurrence of hypoglycemic events within the training data. Another model for BG levels prediction, as described by Plis et al. [23], applied a Kalman filter to CGM data to generate features, which were then utilized by a Support Vector Regression (SVR) algorithm. The algorithm depended on past and current values of insulin, carbohydrates intake and BG levels to estimate future values. The root mean square error (RMSE) values obtained were 22.6 mg/dL for a prediction horizon (PH) of 30 minutes and 35.8 mg/dL for a prediction horizon of 60 minutes. In a similar study [24], a SVR method was also used, but unlike that of Plis, it was only based on CGM data of twelve Type 1 Diabetes (T1D) patients and the results were improved by adding Differential Evolution (DE) algorithms. The results were better than Plis, with RMSE values of 10.78 and 12.95 mg/dL for PH of 30 and 60 minutes, respectively. With the development of Artificial Neural Networks (ANNs), researchers started to use them in the BG prediction domain. Pérez-Gandía et al. [25] relied on CGM data of thirteen T1D patients and proposed a method based on ANN. Their method did not improve the results of those which used classic ML models, and achieved RMSE values of 17.45 and 25.08 mg/dL for PH of 30 and 45 minutes, respectively. In other studies, [26, 27] the approach for predicting BG levels included deep learning methods using Recurrent Neural Network (RNN) based on Long Short-Term Memory (LSTM). The authors shows that the using of LSTM models, which suitable for sequential data such as time series, achieved better results than previous models. While many prior studies have focused on crafting personalized models, a general model would be more flexible and helpful in real clinical situations, especially when patient data availability is limited. In the study by Cobelli et al. [4], patients’ recordings of insulin dosages and meals were utilized by a multi-layer convolutional Neural Network (NN) to learn a generalized model. In [28], a LSTM model is trained using multi-patient BG profiles. Bosoni et al. [29] presented a multi-patient and multivariate deep learning approach, based on LSTM that used flash glucose monitoring (FGM), heart rate (HR), sleep, and physical activity. This approach exhibited superior performance compared to models relying solely on blood glucose (BG) levels.

#### Time-series forecasting

Forecasting stands out as one of the most popular and useful methods in time series analysis. This method aims to predict future values of a variable by leveraging its past values, sometimes supplemented with additional variables. The two primary basic techniques of forecasting are Auto Regression and Moving Average. Auto-regression is based on regression models, where the x variable, or explaining variable is the time.

Moving Average is a forecasting technique that is usually used to identify the direction of a trend. It’s based on the average of a certain number of preceding values. Additionally, there exists a more advanced variant known as the weighted moving average (WMA), which assigns specific weights to each of the preceding time point values. However, determination of the weights can be challenging. This task is often accomplished through optimization techniques such as minimizing the average error of the model. Over the past decade, there has been a growing interest in time series forecasting (TSF) [30, 31], Which has led to the development of new methods that incorporate the use of models from the field of deep learning. The most popular DL models are neural networks based on RNN-LSTM [32] and convolutional neural networks (CNNs). Tan et al. [33] studied different methods for time series forecasting tasks and compared existing solutions and adaptions of Time Series Classification (TSC) algorithms. They compared the different algorithms on a novel archive of 19 datasets. The algorithms they compared included classic ML and ensemble models and DL models such as LSTM and CNN (Resnet [34], Inception [35], FCN [36]) and a novel fast and accurate model named Rocket [37]. They showed that the state-of-the-art TSC algorithm Rocket achieves the highest overall accuracy when employed for regression, outperforming other models. Another notable TSF model is Prophet [38], an open-source tool released by Facebook’s Core Data Science team. Prophet utilizes an additive model that decomposes the time series trends into several components such as seasonality (yearly, weekly, and daily), and also holiday effects. The method’s main advantages include its robustness to shifts in the trend, its ability to handle missing data effectively, and its good handling of outliers. Prophet is particularly well-suited for time series data with strong seasonal effects and multiple seasons of historical data.

## Methods

**Fig 1:**
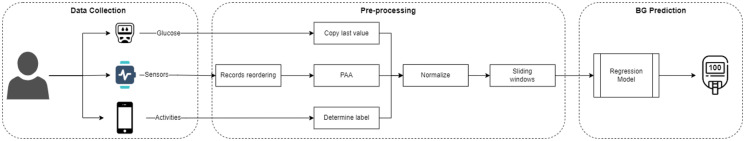
Gludict framework. The overall prediction framework of BG levels, including the collection of the different sources, which are preprocessed and transformed, after which the prediction model is learned and prediction can be made.

We introduce here Gludict, a framework that we had developed for the prediction of glucose levels, that includes the following components—collecting data from several sources, storing the data from all the sources in a database, data transformations, and inducing a prediction model for BG levels. The full framework is presented in Fig 1. In the next sub-sections, we describe each of the components.

### Data sources and collection

The data that was collected for the study and will be required in a real system, consists on three types of data which arrive from three different sources: (1) blood glucose measurements from a Continuous Glucose Monitoring device. (2) a smartwatch device and its sensors’ measurements, which include heart rate, step detector and some motion sensors, and (3) user activities, such as eating or drinking, which the user documents manually using a dedicated app installed on their smartphone.

The dataset included 12 participants along 10 days. They were asked to wear the “Mobvoi TicWatch Pro 2020” smartwatch on their right hand, the DEXCOM G6 CGM device, and their android smartphone. In addition, the activity types and duration were manually recorded by the participants each time they ate or drank using the “GlucoDataSaver” app. The smartwatch application was user-friendly, featuring a limited number of options and requiring minimal daily interaction from users. Participants were prompted once a day to press a button, triggering an upload of their data to the database, ensuring separate storage files for each day. This process helped us track and validate the trial effectively.

The smartphone application was also user-friendly, but participants found manually recording their activities burdensome. Consequently, many activities went unrecorded, which inevitably affected the future results.

The described experiment was approved by Human Subjects Research Committee of Ben-Gurion University. The recruitment period for this study spanned from October 26, 2021, to February 7, 2022. Each participant signed a consent document for the experiment which described the course of the experiment and its purpose. The participants were mostly students—9 of 12, with age range between 22 and 63. The weight range was between 53 and 87 kg. The height range in centimeters was between 162 and 185. The gender distribution was 5 females and 7 males. One of the participants was a T1D.

#### Smartwatch’s sensors’ measurements

To collect physiological measurements of a user, such as hand movements, heart rate, etc., a smartwatch sensors are used. In our study we used the “Mobvoi TicWatch Pro 2020” smartwatch, for which a dedicated Wear OS-based application called “TicWatchSensorReader” was developed, which enables us to extract the relevant sensors. Wear OS, previously known as Android Wear, is a Google’s Android operating system specifically tailored for smartwatches and various other wearable devices. The Wear OS applications are run directly on a smartwatch, given access to the smartwatch’s hardware sensors. For our study, an Android Foreground Service which runs in the background, as long as the smartwatch is kept on hand, was developed. When new data is observed in any of the sensors, the service stores it in the smartwatch’s internal storage, so the data can be accessed directly in the smartwatch or be exported to a smartphone or a computer. Since the service simply documents a measurement when there is a change, the measurements are measured irregularly—in the next section we will describe how we resolved this issue. The measured data is stored on the watch’s memory and can be uploaded via Bluetooth to a smartphone or via Wi-Fi to the cloud, sent by an e-mail, or to a dedicated server. The following four categories of sensors were sampled: 1. Body Sensor: Heart Rate (HR) in units of beats per minute. 2. Activity Sensor: Step Detector which triggers an event each time a step is taken by the user. 3. Motion Sensors: a. Accelerometer: measures the acceleration applied to the device’s x, y, and z axes, including the force of gravity. b. Gyroscope: measures the rate of rotation in rad/s around a device’s x, y, and z axes of a device. c. Gravity: The gravity sensor provides a three-dimensional vector, which indicates both the direction and magnitude of the phone’s orientation relative to gravity. This sensor is commonly utilized to determine the device’s relative orientation in space. 4. Position Sensor: A geomagnetic field sensor that enables the monitoring of changes in the Earth’s magnetic field.

#### CGM based BG measurements

In order to measure the BG levels, we used a “Dexcom G6” CGM device that measures glucose levels continuously and at a constant rate of one measurement every 5 minutes (which is the highest frequency in the market) for about 10 days. The measurements history is stored on the device memory, containing the last three hours. In order to save the entire measurements history, the user is required to download the “Dexcom” application to their smartphone and to pair the smartphone with the CGM device via Bluetooth. The measurements are transmitted automatically to the smartphone using Bluetooth and is also uploaded to DEXCOM’s dedicated server from the smartphone. The measurements history can be viewed on their app or server. The information on the server can be accessed and exported through the DEXCOM’s web interface, which allows it to be further downloaded.

#### Smartphone user activities

**Fig 2:**
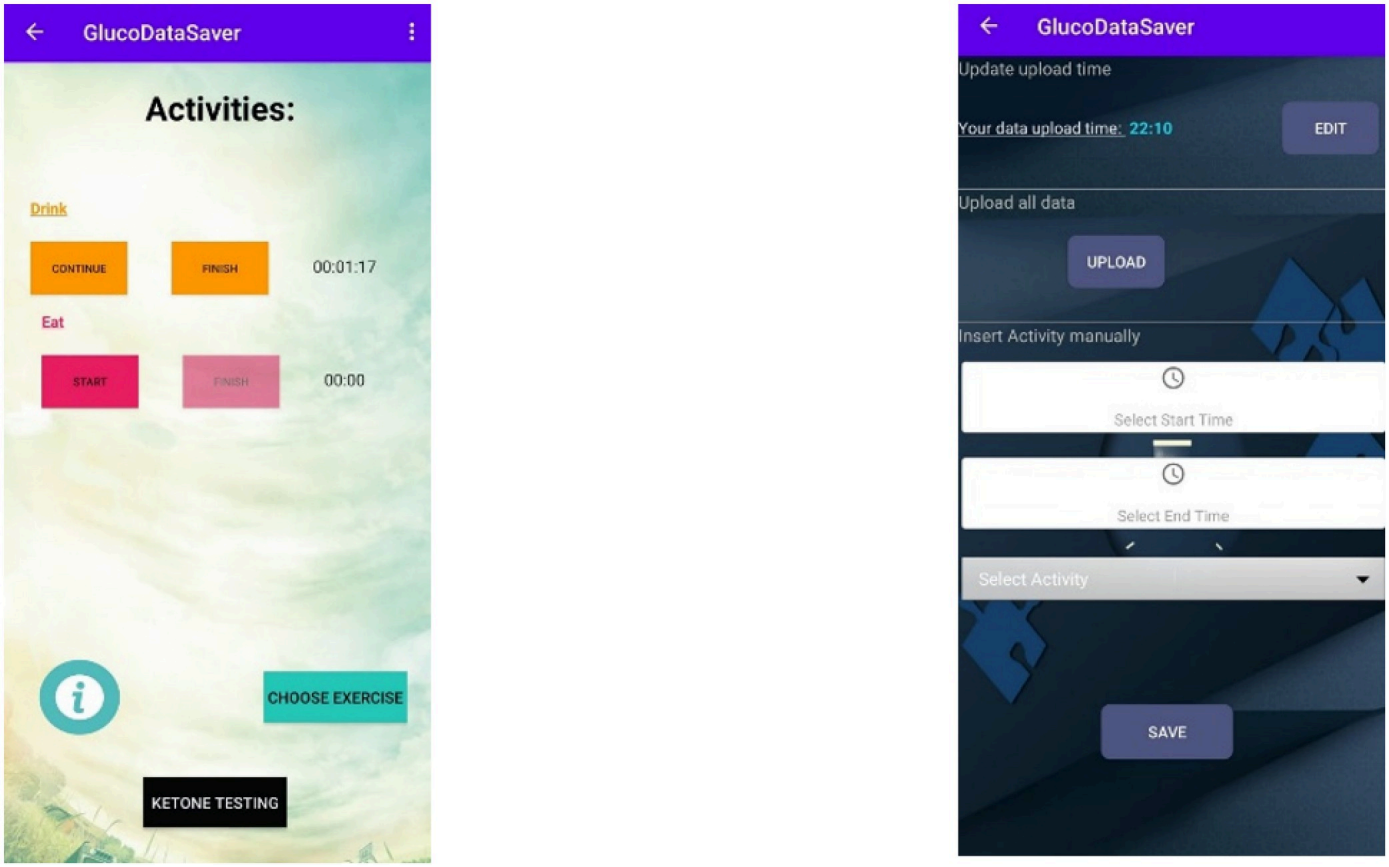
GlucoDataServer app. Screenshots of the android application “GlucoDataSaver”. The right screenshot represents the main screen, and the left screenshot represents the config and “documenting after the fact” screen.

To collect the activities performed by the user (eating, drinking, etc.), we developed an Android-based smartphone application, called “GlucoDataSaver”, that allows the user to manually record his activities. The application includes screens that allow the user to choose activity by type and to save it as a record which includes the start time and end time. For example, when the user wants to document eating activity, he opens the application and presses the “Eating” button. At that moment, the start time and the activity type are saved, and the user is shown a “Eating” screen mode which contains the duration time and “Finish” button. Once the user has finished to eat, he presses the “Finish” button the application return to the main screen and the activity is saved. In addition, the application also allows the user to document activity after the fact, meaning the user can manually document the activity in case he forgot to document it. Fig 2 presents the two main screens of the application. The data is stored on the smartphone memory and in addition, the data can be sent via Wi-Fi to a cloud, e-mail, or dedicated server.

### Data storage

**Fig 3:**
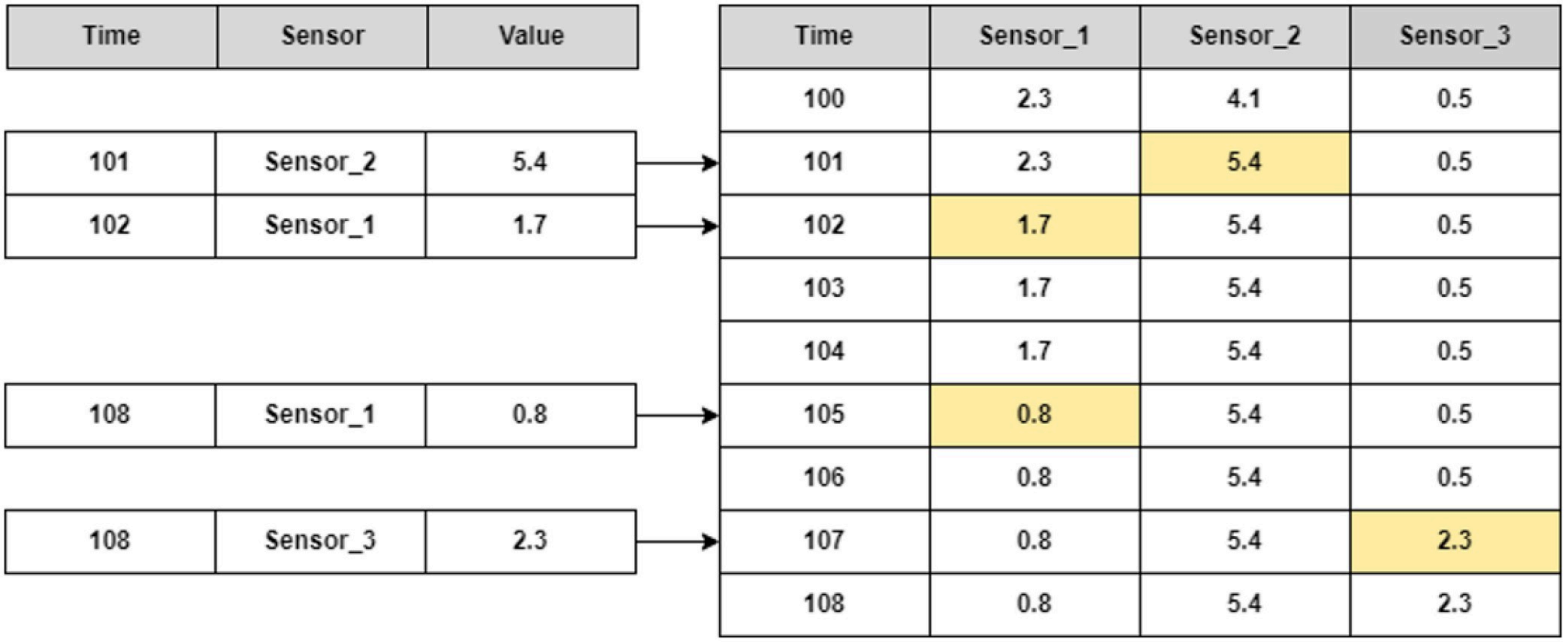
Glucdict’s data storage. The process of consolidating the sensors measurements. On the left side the actual measurements of the device, while on the right there are entire measurements of each time stamp, after filling the values since the last measurement along time.

To be able to analyze the data conveniently we had to store the data in a way that allows fast access and minimal manipulations of the data during retrieval. The activities and CGM device data are already in the proper format (regularly measured), but the sensors data from the smartwatch require some changes, due to the irregular sampling. Thus, as was explained in the previous section, a measurement of a sensor is generated each time the sensor value is changing so each record that contains timestamp and value of only one sensor. To analyze the data, we want each timestamp to include all the sensors’ measurements and not just those that had a change. To achieve this, in each timestamp we copied the sensors values from the previous sample and updated the value of the sensor of the current time stamp measurement, as shown in Fig 3. This mechanism actually created continuous sensors’ data during the periods of use of the smartwatch and phone.

### Predicting BG levels

As mentioned earlier, the data arrives from three different sources, having different sampling rates. The sampling rate of each of the smartwatch’s sensors’ measurements is about every 100 milliseconds, but it is documented only when there is a change in a value of a sensor, while the sampling rate of the BG is every 5 minutes, and the user activities are documented irregularly when an activity occurs. To have a uniformly sampled data, data was imputed in the less granular temporal variables to fit the most frequently sampled variables, which are the smartwatch sensory data, which resulted in a sampling rate of one second. Using the Piecewise Aggregate Approximation (PAA) method [39] the smartwatch variables sampling rate were decreased into a measurement per second, based on the mean value, except the step detector sensor that was represented by the number of steps within a second. The user activity was represented by the activity label that was on within the last second (0—none, 1—eat, 2—drink). To make sure that the model is not influenced by the values range of each of the temporal variables, normalization was used. The data was normalized by Min-Max scaler where the minimal value of the temporal variables is made equal to zero and the maximal to one. The Min-Max Scaler scales the values to a specific value range without altering the shape of the original distribution.

#### Predicting blood glucose values a prediction time ahead

**Fig 4:**
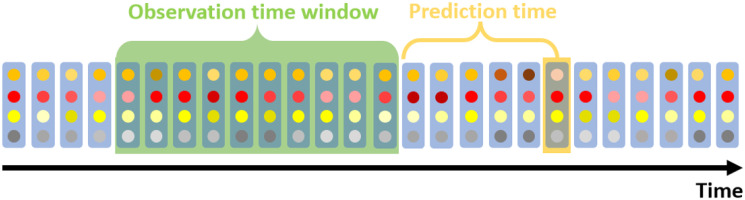
Sliding window structure. Illustration of the multiple temporal variables along time, in which each horizontal sequence of dots represents a temporal variable, and their tones illustrates different values. Based on all the temporal variables in the observation time period, a value in a prediction time period ahead is predicted.

Using the data from the previous step, a sliding observation time window was used, based on which data a value of the blood glucose was predicted a prediction time ahead, as illustrated in Fig 4. This mechanism eliminates time dependence and creates independent continuous windows. It is very important since the smartwatch’s and phone’s sensors were monitored only during the day.

#### Prediction modelling

We describe here briefly the prediction models that were used as part of the method for the blood glucose forecasting.

#### Rocket

Rocket [37] is a time series classification and regression method. Rocket’s primary advantage is achievement of high accuracy with low runtime. It transforms the data using random convolutional kernels, such as those used in a CNN, by projecting it into a higher dimensional space. These kernels were shown to capture relevant features such as shape, frequency or variance without requiring handcrafted features. The transformed features are subsequently inputted into a linear classifier, such as Ridge Regression. This method operates at high speed since it bypasses the need to learn kernels, unlike deep learning methods.

#### Prophet

Additional model we used as a baseline in our research is the Prophet [38]. Prophet is an open-source software released by Facebook’s Core Data Science team. This forecasting approach uses an additive model to analyze time series data, breaking down trends into several components, including seasonality (yearly, weekly, and daily) and holiday effects. Prophet demonstrates robustness in handling missing data and trend shifts, while also exhibiting a capability to effectively manage outliers. The parameters of the Prophet model we used are changepoint_prior_scale, seasonality_prior_scale, holidays_prior_scale and seasonality_mode.

#### LSTM

Another model we tried is a LSTM-based model. LSTM is a type of artificial recurrent neural network (RNN) architecture commonly utilized in the field of deep learning [32]. Unlike standard feedforward neural networks, LSTM has feedback connections. It can process not only individual data points but also entire sequences of data. A LSTM unit comprises a cell, an input gate, an output gate, and a forget gate. LSTM networks are highly suitable for classifying, processing, and predicting based on time series data. This is because they can effectively handle situations where there are lags of unknown duration between significant events in a time series.

#### ResNet

Additional model is Residual Networks (ResNets) [34], a deep convolutional network. Its architecture is designed to support hundreds or thousands of convolutional layers. The fundamental concept involves bypassing blocks of convolutional layers through shortcut connections. These connections allow the network to learn residual mappings instead of attempting to learn direct mappings, which enables the successful training of much deeper networks.

#### Inception

A more recent model is the Inception [35], which represents a substantial advancement over existing deep learning models. It has demonstrated competitive performance compared to other state-of-the-art models. The key innovation of the Inception model is the use of inception modules in Convolutional Neural Networks, which consist of multiple parallel convolutional layers of different kernel sizes. The inception modules were devised to address both the issues of computational expense and overfitting.

## Evaluation

### Research questions

For this study, we formulated four primary research questions, each accompanied by corresponding experiments designed to address them. The main research questions were:

1. What is the best observation time window duration for each of the prediction time periods?
2. Does BG levels prediction using user activities data from a smartwatch, in addition to CGM data, performs better than solely CGM data?
3. Does BG levels prediction using a smartwatch’s sensors data, in addition to CGM data, performs better than solely CGM data?
4. Which of the smartwatch’s sensors, or their combination, in addition to the glucose measurements, or without, leads to the best BG prediction?

#### Models parameters and baselines

To run the prediction models that were described before, it is required to tune the model’s parameters. Each model type has different tuning parameters that were used: for the DL models—LSTMs, Resnet, Inception—we used the parameters: number of epochs, batch size and learning rate. For the Prophet model we used the parameters: changepoint_prior_scale, seasonality_prior_scale, holidays_prior_scale and seasonality_mode, and for the Rocket model we used the parameter: number of kernels.

Each model was trained and tested on each participant separately. Each participant had a large dataset of sliding windows. A nested cross validation was used to get appropriate results and for hyper-parameter tuning.

### Experimental plan

To answer the research questions, three experiments were designed. We trained a 3D structure (a tensor representation), from the sensors and glucose data, as we described in the previous sections, for Resnet, Inception, RNN-LSTMs, Prophet, and Rocket models.

#### Experiment 1—Observation time duration

The goal of this experiment was to evaluate the best observation time windows’ duration (research question 1). For that 30, 60 or 90 minutes durations of observation time windows were used, including: glucose measurements, heart rate, step detector, activities, and the motion sensors. The observation time durations were experimented with of 5, 10, 15, 30, 45 or 60 minutes prediction time periods. We applied the models: Resnet, Inception, RNN-LSTMs, Prophet, and Rocket, with all the temporal variables combinations, and separately, and different model’s parameters, such as batch size, epochs, learning rate for the ANN models and number of kernels for the Rocket model.

#### Experiment 2—Best prediction model

To answer the rest research questions, we would like to find which model achieves the best results. For that, we performed the second experiment which focused on evaluating the performance of the prediction models—Resnet, Inception, RNN-LSTMs, Prophet, and Rocket—to find out the best model based on a specific observation time window. For that the best observation time window, which performed best in experiment 1 was intended to be used. For each model we evaluated the best model’s parameters by applying each model with different model’s parameters, and temporal variables combinations as described in experiment 1.

#### Experiment 3—Best temporal variables combinations

This experiment focused on evaluating the best prediction results and which temporal variables combinations achieve those results (research questions 4). Since we tested all combinations of the temporal variables, we can also answer the research questions 2, 3. For that the best observation time window, which evaluated in experiment 1, and the best model which evaluated in experiment 2 were used.

#### Experiment 4—Comparing our method to previous methods

This experiment focused on evaluating our method against previous methods in the literature. Because our dataset is unique, it is not true to compare our results (RMSE) to previous articles results, so we applied previous methods on our dataset and compared the results. The article we found as the best for comparing is the article of Bosoni et al. [29], whose method achieved better results compared to other methods on the same datasets. In addition, their method and experiments are clearly described so that we could reproduce the method accurately. The temporal variables we used for their method are glucose and HR, as they did in the article. The results of the Bosoni et al. [29] we compared to our best results, as we found in experiment 3.

#### Evaluation metrics

The metrics we used to evaluate the results are Root Mean Square Error(RMSE) and Clarke Error Grid Analysis (EGA).

RMSE effectively highlights the magnitude of prediction errors, including the impact of larger deviations, while Clarke EGA provides a critical assessment of the clinical implications of those errors visually. Their combination ensures that the model’s predictions are both numerically accurate and safe for patient care.

#### Root Mean Square Error (RMSE)

RMSE is a commonly used metric for evaluating the performance of a regression model. It is the standard deviation of the residuals (prediction errors). Residuals are the differences between the observed and predicted values. They measure how far from the regression line data points are. The RMSE formula contains the predicted value ($\hat{y}$), the observed value (y) and the number of observations (n).

$$\begin{array}{c} RMSE=\sqrt{\sum_{i=1}^{n}\frac{{\left({\hat{y}}_{i}-y_{i}\right)}^{2}}{n}} \end{array}$$

#### Clarke Error Grid Analysis (EGA)

The EGA was introduced by Clarke et al. in 1987 [40] and is widely used in the evaluation of glucose monitoring systems. The metric evaluates the differences between estimated and actual BG levels over a specified time horizon. These differences are visually represented on a chart. EGA uses a grid with five zones, each represents a different level of clinical significance in terms of the impact on patient management. Zone A is defined as clinically accurate. It includes predicted values that have a deviation of no more than 20% from the real values, or values falling within the hypoglycemic range (<70 mg/dL). In Zone B, the differences between the predicted and real measurements exceed 20%, but it causes little or no harm to the patient, so it’s still acceptable. Zone C represents significant errors that could lead to unnecessary treatment changes. In Zone D, instances signify a failure to detect and address deviations in blood glucose levels. This occurs when the actual blood glucose levels fall outside the acceptable range, despite the predictions being within acceptable limits. Zone E identifies instances where predicted values contradict real or measured blood glucose levels, potentially leading to treatments contrary to recommended actions.

## Results

We describe here the results of the experiments that were described earlier.

### Experiment 1—Observation time duration

**Fig 5:**
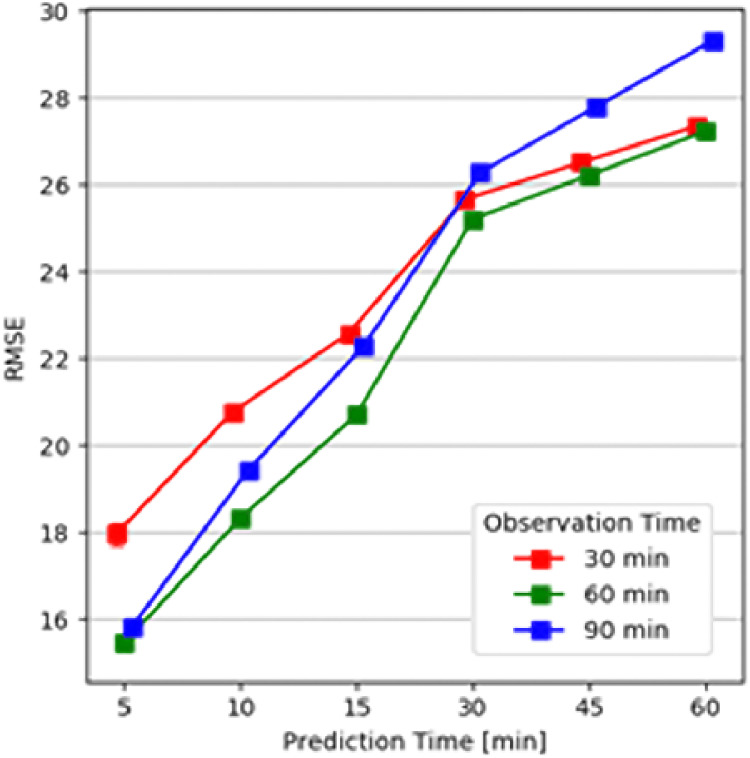
Average RMSE over different time durations. A sixty minutes observation time window outperforms for any of the prediction time periods ahead.

In the first experiment, the goal was to determine which observation time window size performs best for each prediction period ahead. Therefore, all models were evaluated with all possible temporal variables combinations and an observation time window of 30, 60, and 90 minutes. Fig 5 shows the mean prediction results when using each of the three observation time windows to predict a prediction time period ahead of 5, 10, 15, 30, 45, 60. While the use of a ninety minutes observation time window performed better than the use of thirty minutes to predict up to 15 minutes ahead, it was the opposite for longer prediction time periods ahead. However, the use of a sixty minutes observation time performed best for any of the prediction time periods ahead.

### Experiment 2—Best prediction model

**Fig 6:**
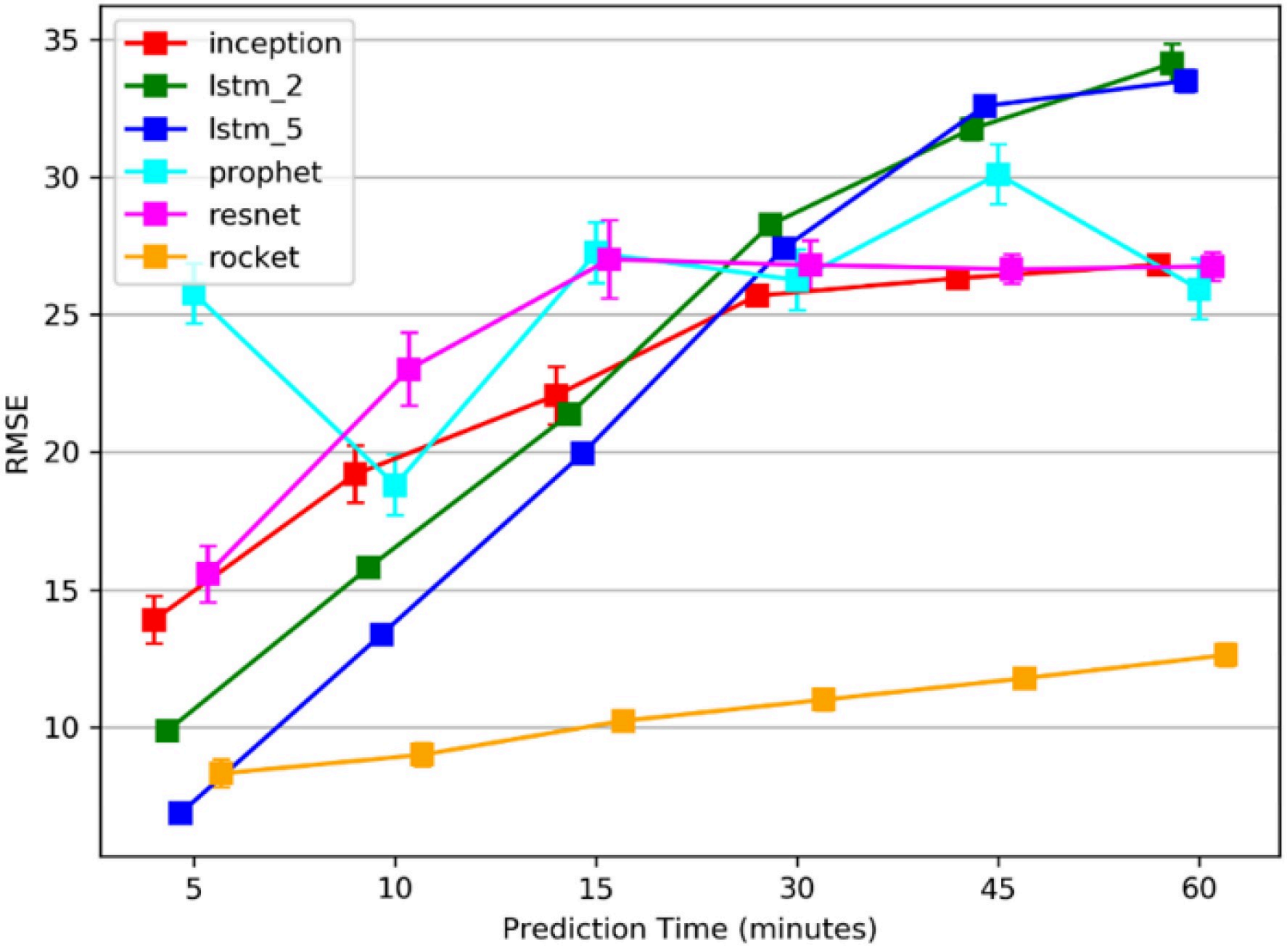
Average RMSE comparison between models. RMSE results show that Rocket outperformed other models while using a 60 minutes observation time window.

In this experiment the focus was on the models’ performance. Since the use of a sixty minutes observation time performed best in experiment 1 for all the prediction time periods, we report the models’ performance on the use of this observation time window. Fig 6 shows the mean results of the various prediction models for each of the prediction time periods ahead. The Rocket, with 100000 kernels achieved the best performance compared to the use of less kernels that were tested: 100, 1000, 10000, 200000, and performed significantly better than any of the other models for all the prediction times periods.

### Experiment 3—Best temporal variables (sensors) combination

**Fig 7:**
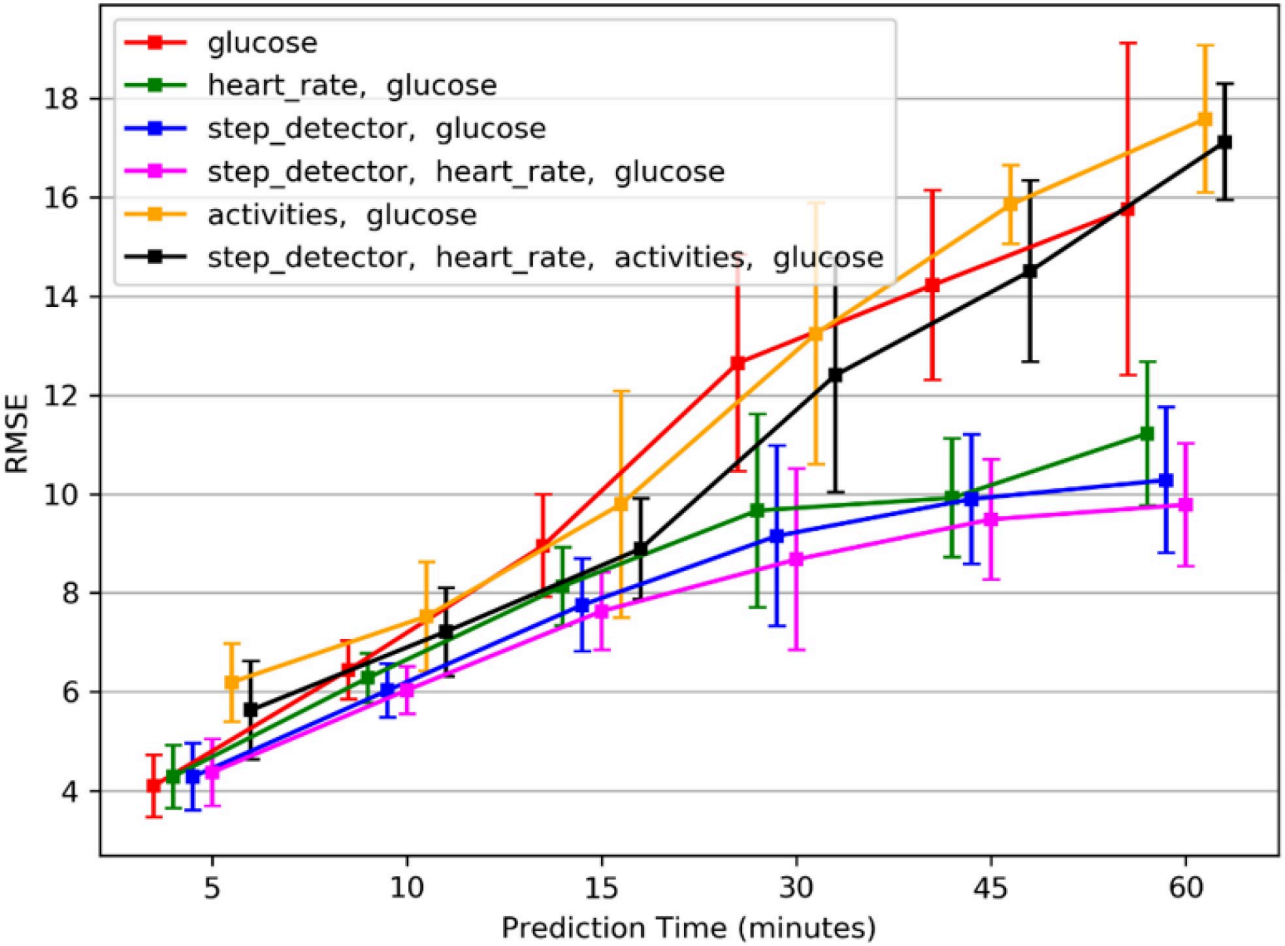
Average RMSE comparison between temporal variables combinations. A combination of glucose, heart rate and steps count using Rocket model with 60 minutes observation time window has the best performance.

In this experiment the focus was on identifying the best performance and the parameters that lead to it, as well as the best temporal variables combination. Since the use of a sixty minutes observation time performed best in experiment 1, and the Rocket model preformed best in experiment 2, we report the results when using the sixty minutes observation time period and the Rocket model. Fig 7 shows the mean results of the temporal variables combinations for each of the prediction time periods. Using glucose solely, and glucose together with activities performs worse, while with additional temporal variables the performance was improved. The use of additional temporal variables together with the glucose performs significantly better than when consisting only on glucose, for all prediction times periods except 5 minutes in which all the options perform similarly. The glucose together with the heart rate and the step detector combination performs best.

### Experiment 4—Comparing our method to previous methods

**Fig 8:**
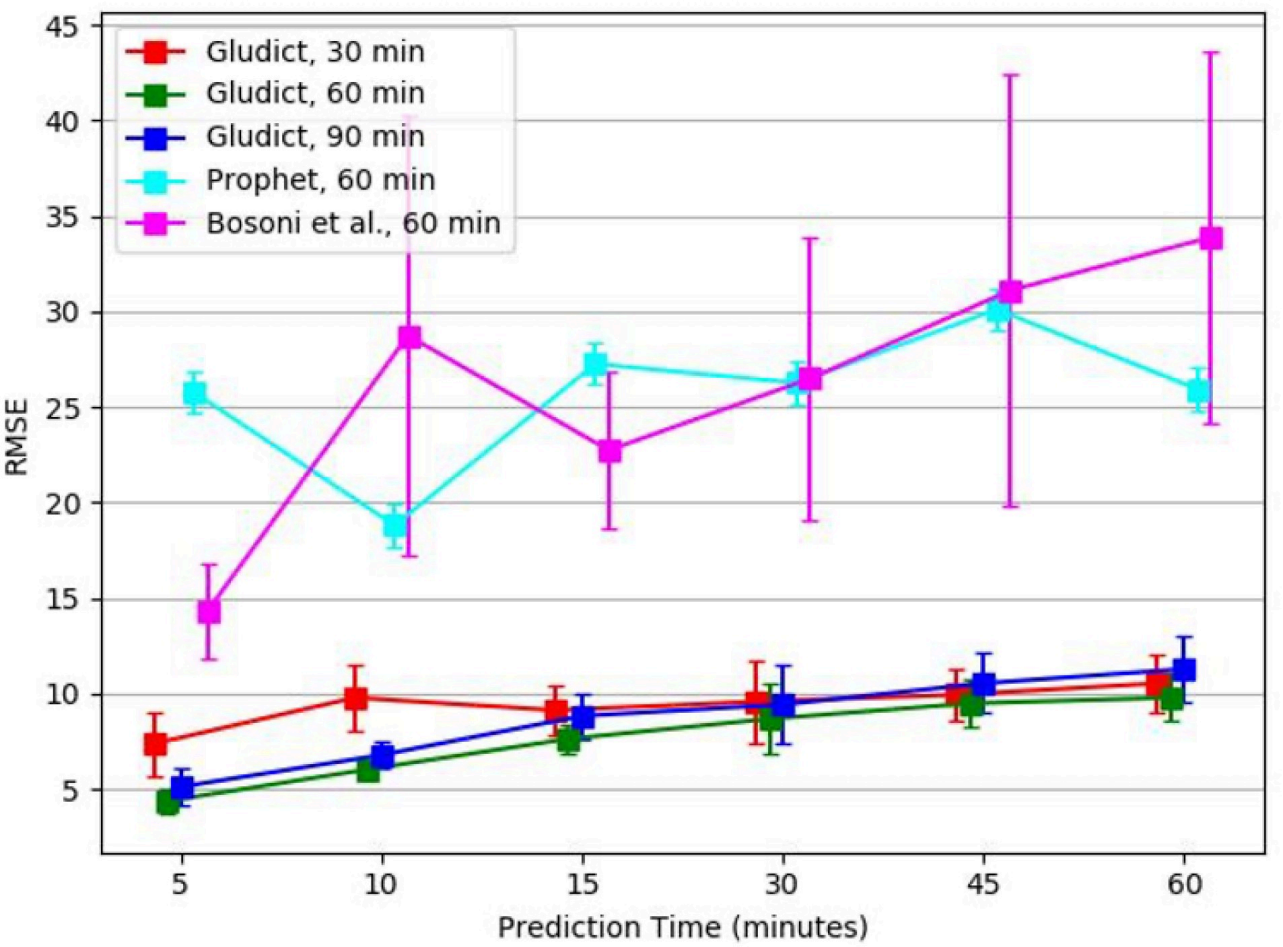
Comparison to previous methods. Gludict that uses the Rocket model with the best variables combination performs significantly better than the use of Prophet, or Bosoni’s method.

Fig 8 shows the mean prediction results of the best temporal variables combination of glucose, HR and step detector—when using each of the three observation time windows of 30, 60 or 90 minutes, to predict a prediction time period ahead. While the use of a ninety minutes observation time performed better than the use of 30 minutes to predict up to 30 minutes ahead, it was the opposite for longer prediction time periods ahead. However, the use of a sixty minutes observation time performed best for any of the prediction time periods ahead. Additionally, we show here for comparison two baselines, the methods that was presented by Bosoni et al. [29] and when we used the Prophet method both with 60 minutes observation time period, which worked best for each of them. As can be seen Gludict performed best and even significantly.

**Fig 9:**
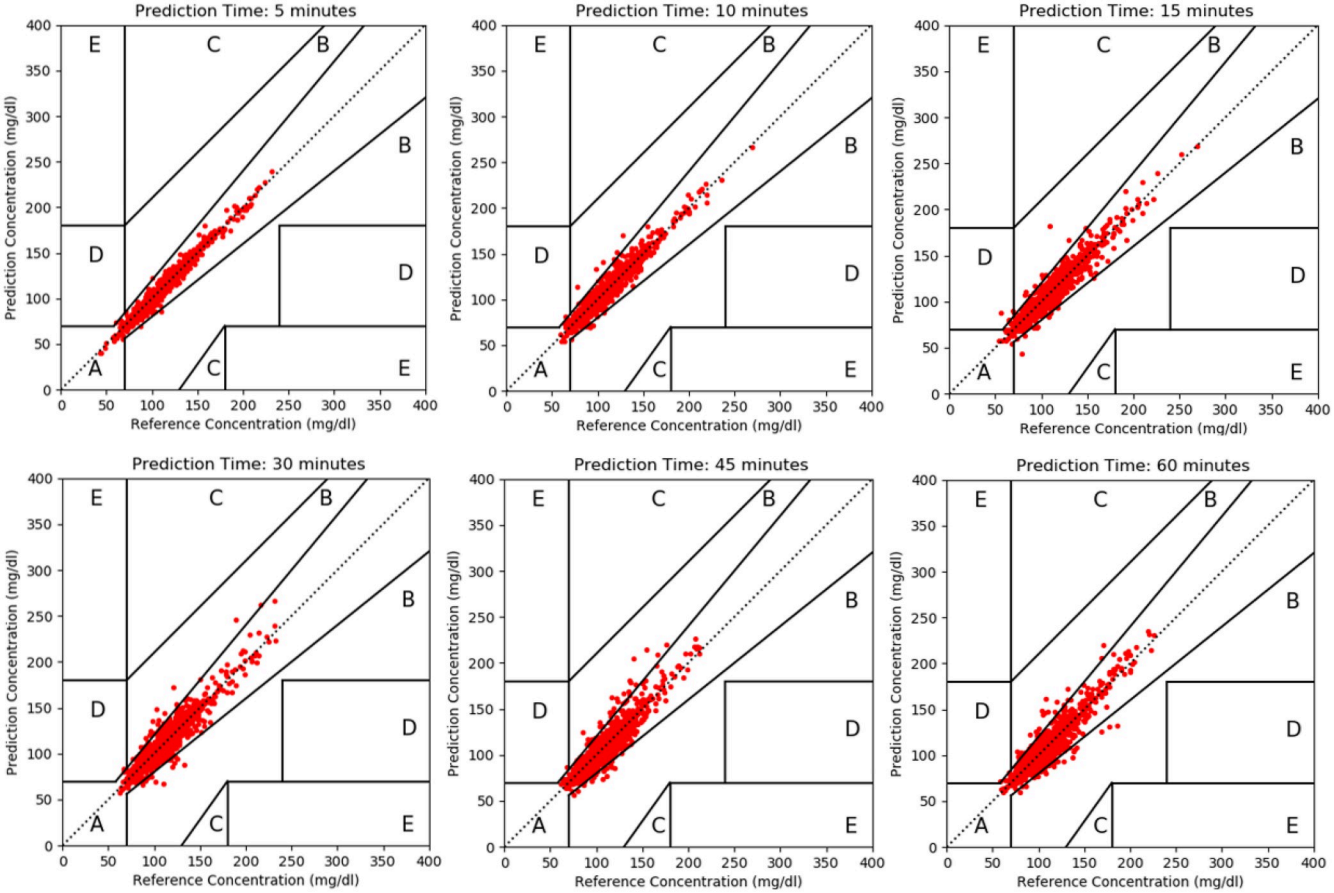
Clarke error grid analysis. Gludict that uses the Rocket model with the best variables combination performs well on different prediction times.

Fig 9 shows the results of each prediction time on the Clarke Error Grid Analysis, which gives an illustration of the prediction accuracy in clinical terms, as was explained in the subsection on evaluation metrics. Most of the predictions are in region A, which is considered clinically safe. There are also few predictions that are in region B, which is still considered uncritical. In prediction time of 15 minutes ahead there is only one prediction that are in region D, which is obviously meaningless and can be a result of noise. According to the grids we can see that our method predicts the blood glucose accurately and more importantly, is clinically safe.

## Discussion and conclusions

Predicting BG levels is crucial for a variety of purposes, including the management of type 1 diabetes and the optimization of performance for professional athletes. BG levels are influenced by daily activities such as eating and drinking, varying according to their contents, as well as by exercise. BG levels that deviate from the normal range can lead to both continuous and temporary life-threatening medical complications. Anticipating near-future blood glucose (BG) levels, even just minutes ahead, allows for the early identification of abnormal values. This proactive approach enables interventions aimed at averting hypo.

hyper-glycemia and their concomitant complications. In this paper, we proposed a BG prediction framework that utilizes data from smartwatch sensors and user activities, in conjunction with continuous data from a CGM device. Our goal was to build a model capable of forecasting blood glucose (BG) levels for time intervals of 5, 10, 15, 30, 45, and 60 minutes in advance. We hypothesized that incorporating data from smartwatch sensors and user activities would enhance the predictive performance of the model.

For that a whole framework was implemented, including an agent in the smartwatch, and a mobile app for the user activities documentation. We had performed a rigorous evaluation of the framework applying several settings and prediction models, in which we found that using an observation time window of 60 minutes performed best, as well as the Rocket model according to the RMSE index. Additionally, we had found that the use of heart rate and step detector in addition to the CGM data performed best achieving an RMSE between 4-10 mg/dl depending on the time ahead. According to the EGA, our method predicted the BG levels accurately and clinically safe. Furthermore, our findings indicated that incorporating user activities did not enhance prediction accuracy and, in fact, resulted in poorer performance compared to using glucose as a single feature. Thus, even with an automatic method capturing the eating/drinking activities of a user, it wouldn’t necessarily improve forecasting performance. This is likely because it’s uncertain whether the contents include carbohydrates that raise BG levels.

Our study consists 12 participants and only one diabetic patient. The sample size is not that big due to the costs, but since it is over a period of ten days, and since the purpose is to be able to predict based on single subject, the amount of data is quite meaningful. The aim of this study is to predict glucose levels in general, without specifically focusing on diabetic patients. The model behaved similarly to all of the patients, including the one with diabetes. In the future we would like to collect data from a dozen of type one and two diabetic patients in order to demonstrate that the model behave similarly.

In experiment 4, we compared our best model to Bosoni et al. [29], whose method achieved better results compared to other methods on same datasets. Our method achieved RMSE in the range of 4-10 mg/dl for different prediction horizons (PH), while Bosoni et al. had RMSE in range of 11-17 mg/dl for 5 minutes PH and 17-44 md/dl for other PH on our dataset. In addition to the large gap in RMSE, Bosoni et al. and many others used RNNs ans LSTMs as their model, while our best model was Rocket which is also much faster.

In conclusion, we highly recommend using physiological data like step count and heart rate alongside the glucose for better prediction. In addition, using automatically monitored data brings much better results than using manually recorded activities.
